# Supplementary figures and images for: Genome-Wide Analysis of the WOX Family and Its Expression Pattern in Root Development of Paeonia ostii
Source: Int J Mol Sci. 2024 Jul 12;25(14):7668. doi: 10.3390/ijms25147668 (PMC11277081; doi:10.3390/ijms25147668)

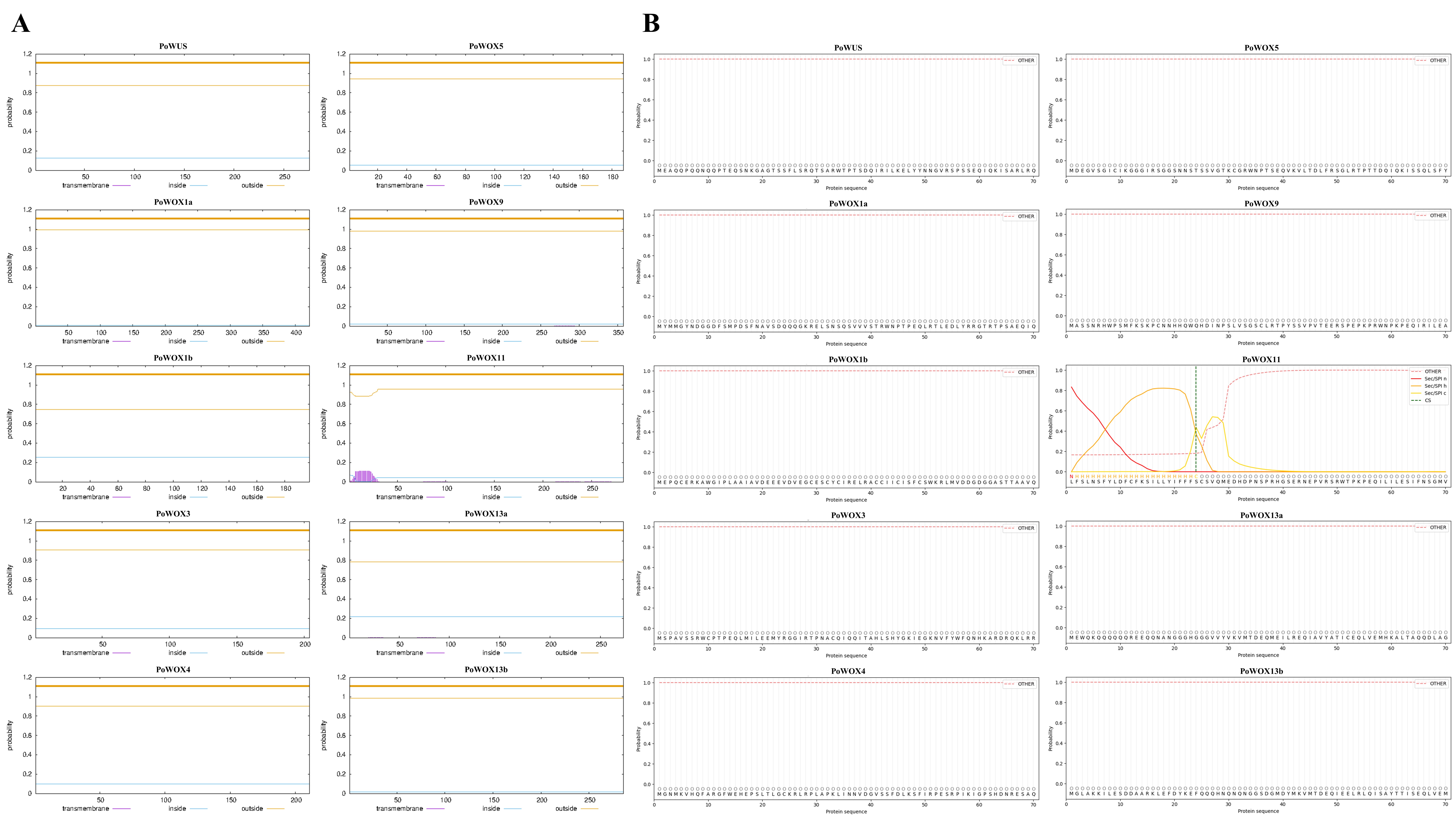

Supplement: Supplementary file 1 [file ijms-25-07668-s001.zip › Supplementary Materials/Figure S1.jpg]
